# Rapid-Eye-Movement-Sleep (REM) Associated Enhancement of Working Memory Performance after a Daytime Nap

**DOI:** 10.1371/journal.pone.0125752

**Published:** 2015-05-13

**Authors:** Esther Yuet Ying Lau, Mark Lawrence Wong, Kristy Nga Ting Lau, Florence Wai Ying Hui, Chia-huei Tseng

**Affiliations:** 1 Sleep Laboratory, The University of Hong Kong, Hong Kong, China; 2 Department of Psychology, The University of Hong Kong, Hong Kong, China; 3 Department of Psychiatry, The University of Hong Kong, Hong Kong, China; Oasi Research Institute, ITALY

## Abstract

The main objective was to study the impact of a daytime sleep opportunity on working memory and the mechanism behind such impact. This study adopted an experimental design in a sleep research laboratory. Eighty healthy college students (Age:17-23, 36 males) were randomized to either have a polysomnography-monitored daytime sleep opportunity (Nap-group, n=40) or stay awake (Wake-group, n=40) between the two assessment sessions. All participants completed a sleep diary and wore an actigraph-watch for 5 days before and one day after the assessment sessions. They completed the state-measurement of sleepiness and affect, in addition to a psychomotor vigilance test and a working memory task before and after the nap/wake sessions. The two groups did not differ in their sleep characteristics prior to and after the lab visit. The Nap-group had higher accuracy on the working memory task, fewer lapses on the psychomotor vigilance test and lower state-sleepiness than the Wake-group. Within the Nap-group, working memory accuracy was positively correlated with duration of rapid eye movement sleep (REM) and total sleep time during the nap. Our findings suggested that “sleep gain” during a daytime sleep opportunity had significant positive impact on working memory performance, without affecting subsequent nighttime sleep in young adult, and such impact was associated with the duration of REM. While REM abnormality has long been noted in pathological conditions (e.g. depression), which are also presented with cognitive dysfunctions (e.g. working memory deficits), this was the first evidence showing working memory enhancement associated with REM in daytime napping in college students, who likely had habitual short sleep duration but were otherwise generally healthy.

## Introduction

Sleep is found to play an important role in human cognitive functions, including sustained attention and vigilance [1], memory [2], emotion processing [3], as well as working memory [4]. While most previous studies investigated the role of sleep in cognitive functioning by looking at the outcomes following sleep loss, an increasing number of studies started to reveal the significance of sleep in cognitive functioning by providing participants with an extra daytime sleep opportunity (i.e. a daytime nap). For instance, among non-sleep-restricted adults, a daytime nap has been found to improve individuals’ vigilance [5], declarative [6] and procedural memory [7], as well as emotional processing [8]. However, existing napping studies rarely addressed sleep’s role on working memory ability [9], which has been consistently found to be closely related to sleep quantity/quality by studies using a sleep deprivation/restriction paradigm [4] or of a sleep-disordered patient sample (e.g. insomnia [10] or obstructive sleep apnea [11]).

Subserved by the prefrontal cortex (PFC) and related networks, working memory is considered as the set of systems necessary to keep things in mind while performing complex tasks, such as reasoning, comprehension and learning [12,13], and it has been found to be one of the most affected neurocognitive domains across sleep deprivation studies [14]. After 21 hours of sleep deprivation, healthy adults were found to have lower accuracy and slower reaction time on the n-back task, a working memory task, when compared to baseline task performance [15]. The deterioration of working memory ability was further observed to associate with electroencephalographic (EEG) measures, including degraded event-related brain potentials in attention focusing ability. Following 30 hours of sleep deprivation, healthy adults showed worse accuracy and reaction time on working memory tasks, in comparison with those who had normal sleep [16]. Neuroimaging data further revealed decreased global brain activation and decreased activation in the bilateral frontoparietal circuits accompanied with deteriorated working memory performance following sleep deprivation [17].

Apart from total sleep deprivation, studies using chronic sleep restriction paradigm also reported working memory deficits. For example, Van Dongen and coworkers [18] found that individuals with chronic sleep restrictions (6 hours or less for 14 consecutive nights) had deficits in working memory ability comparable with those having two nights of total sleep deprivation. Lo and coworkers [4] found that those under chronic sleep restriction (5.75 hours of sleep time on average for seven consecutive nights) had worse working memory ability than the controls (8.56 hours for the same period) on the n-back tasks. With neuroimaging data, Beebe and coworkers [19] found that compared to those allowed to have 10 hours of sleep for a week, healthy adolescents with chronic partial sleep restriction (6.5 hours of sleep) who maintained comparable working memory task performance at baseline had increased activation in the dorsolateral prefrontal cortex and inferior parietal lobes, which were interpreted as increased compensatory effort for optimal task performance following sleep loss.

The effect of sleep loss on working memory was found to relate to the degraded PFC activation and functional connectivity with other brain regions. During sleep, particular sleep physiology was found to contribute to the optimal functioning of the PFC. Functional imaging data revealed that from wakefulness to non-rapid-eye-movement sleep (NREM), cortical activity in the PFC became increasingly weakened. In rapid-eye-movement sleep (REM), however, increased activation was recorded in the PFC again [20–23]. Also during REM, frontal areas were recorded with increased functional coupling with other brain regions, including the posterior brain [24]. Using near-infrared spectroscopy, a recent napping study found that dorsolateral PFC had increased oxygenated hemoglobin concentration from stage 2 NREM to REM, and the first onset of REM (REM latency) specifically coincided with the activation of the dorsolateral PFC [25]. These neuroimaging data all pointed to a close association between REM with the PFC activation and functional connectivity with other brain regions. However, little was known regarding the association between REM and PFC functioning, in terms of functional significance in adults without sleep pattern manipulations (e.g. sleep deprivation/restriction paradigm) or healthy individuals without sleep disorders (e.g. sleep apnea, REM behavioral disorder or insomnia.)

Collectively, the findings of the existing sleep-deprivation/restriction literature indicated that sleep loss led to deteriorated working memory performance, which was associated with altered activation of the prefrontal cortex (PFC) as well as its functional connectivity with other brain regions. However, little was known about the role of sleep gain in the cognitive functions subserved by the PFC. Indeed, studies addressing the role of daytime sleep opportunity on working memory and other cognitive functions subserved by the PFC were called for [9].

The current study aimed to investigate the role of sleep in working memory ability with the use of a napping paradigm. Given that sleep is essential for optimal PFC functioning and sleep leads to reduced working memory performance, we hypothesized that healthy adults would have improved working memory performance following a daytime sleep opportunity when compared with wakefulness for the same period, as sleep was shown to be essential for optimal PFC functioning. We further hypothesized that the post-nap working memory performance would be related to REM sleep given that REM had been shown to relate to the hemodynamic change of dorsolateral PFC which subserved human working memory ability.

## Methods

Ethics approval has been obtained from the Human Research Ethics Committee for Non-Clinical Faculties by the University of Hong Kong before the commencement of this study. All participants provided written informed consent before they started the protocol. The protocol was prepared in accordance with the Helsinki Declaration.

### Participants

Previous studies of napping effects on cognitive functions mostly had a sample size of 50 (e.g. [8]). To investigate the potential moderating effects of baseline sleep characteristics, such as poor sleep quality, we recruited 90 healthy college students from campus campaign, including mass emails, posters and word of mouth from December 2012 to May 2014. All participants were assessed with the following inclusion criteria 1) aged 17–25, 2) doing a full-time degree at a local university, 3) willing to comply with the study protocol and 4) able to provide written informed consent, and exclusion criteria 1) presence of pre-existing sleep disorders, 2) with severe medical conditions, 3) use of medication within two weeks of the study and 4) use of caffeinated and/or alcoholic beverage within 24 hours before the commencement of the experimental session. Five participants were excluded as they met the exclusion criteria (i.e. pre-existing diagnoses of sleep disorders, severe medical conditions or current use of medications within two weeks of the study). All sessions of data collection were conducted in the Sleep Laboratory of the University of Hong Kong from December 2012 to May 2014.

### Procedures

The current study was part of a larger study examining the relationships among nighttime sleep, daytime sleep opportunity and neuropsychological functioning in young adults.

#### Experimental design

The study had a 7-day protocol. On the first day, after the consenting procedure and screening, participants completed questionnaires on sleep and mood and were instructed to fill out the sleep diary and use the actigraph-watch (see Measurements for details) throughout the 7-day protocol. Participants were asked to follow their habitual sleep-wake behaviors throughout the study protocol.

On the sixth day, participants returned to the laboratory for a half-day experimental session, without any prior napping or consumption of caffeinated/alcoholic beverage on that day. All sessions started at 1pm (pre-test). The participants first completed state-measurements on sleepiness and affect, followed by neurocognitive measures (including vigilance, attention and working memory, see Measurements for details), which in total took about 1 hour. They were then randomized (simple randomization with an 1:1 allocation ratio) to either have a daytime sleep opportunity or remain wakeful in the laboratory. Sequentially numbered envelopes were prepared and sealed before recruitment of participants and were opened consecutively after each eligible participant consented to participate in the study by the coauthor (WML). In the Nap-group, participants were allowed to sleep for about 90 minutes for a normal sleep cycle, monitored by polysomnography (E-series Compumedics Ltd, Abbotsford, Victoria, Australia). In the Wake-group, participants were asked to follow their usual routine, except that no exercise was allowed. Most participants spent the time on using electronic devices (e.g., Internet browsing) or academic work. At 5:30pm, the participants completed another session of state measurement and neurocognitive measures (post-test). To monitor any potential effects of the nap on subsequent nighttime sleep, participants completed the sleep diary and wore the actigraph-watch for one more day and returned both items on the seventh day, followed by debriefing.

#### Sleep recording

The EEG sites included C3, F3 and O1 (referenced to A2) and C4 (referenced to A1). The sleep stages were scored with reference to the guidelines of the American Academy of Sleep Medicine [26] by a registered sleep technologist blind to the study aims and participants’ task performance. The polysomonographic measures included total sleep time, sleep onset latency, waking time, duration and percentage of NREM stages 1–4 (with stage 3 and 4 NREM combined as SWS), REM and REM latency.

### Measurements

All questionnaires were written in Chinese language. For sleep measurements, participants completed a sleep diary to report their total sleep time for the whole 7-day protocol. The total sleep time (TST) was calculated by subtracting the sleep onset latency (SOL), wake after sleep onset (WASO) from the period between they went to bed and woke up. Participants were also asked to jot down their use of medication, alcohol, caffeinated beverage as well as daytime napping behavior in the sleep diary. They also wore an actigraph-watch (Micro Motionlogger Sleep Watches, Ambulatory Monitoring, Inc.) on their non-dominant hand for objective measurement of sleep-wake patterns throughout the 7-day protocol. The actigraph-watch was set at 1-minute bins in zero crossing mode. Sleep-wake cycles were analyzed with the Action 4 software (Ambulatory Monitoring Inc.). The sleep onset, offset, and total sleep period were estimated following a validated algorithm [27].

In addition to objective measures, self-reported measures on sleep quality and daytime sleepiness were also used. Participants completed the Pittsburgh Sleep Quality index (PSQI) [28, 29] for their sleep quality over the previous month and the Epworth Sleepiness Scale(ESS) [30,31] for daytime sleepiness. Poor sleep quality was defined by a PSQI global score >5 and excessive daytime sleepiness by an ESS score >1. Participants also completed the Stanford Sleepiness Scale (SSS) [32] at the beginning of the pretest and posttest as a measure of their state-sleepiness.

For mood measures, participants completed the Depression Anxiety Stress Scale 21-item (DASS) [33, 34] to report their negative mood symptoms for the previous week.

The Positive and Negative Affect Schedule (PANAS) [35] was translated into Chinese language with back translation by independent bilingual speakers and used as a measure of their state-positive/negative affect.

For neurocognitive measures, participants completed the Psychomotor Vigilance Test as a vigilance measure. The palm version of PVT was administered (downloaded from www.corware.com) [36, 37] in which participants had to respond as quickly as possible to a stimulus appearing on the screen by pressing a button. The test duration was about five minutes. The average response time, 1/average response time (reciprocal transform, operationalized as dividing each RT by 1000 and reciprocally transformed) and number of lapses (omission errors, operationalized as RTs > 500ms) were recorded. The N-back task was used to measure their basic attention (0-back condition) and working memory (2-back condition). In the n-back task [38], participants were presented a symbol appearing at a randomly selected location amongst 12 possible positions. In the 2-back condition, participants had to decide whether each symbol matched the position of the symbol presented two items ago. There were three experimental blocks and in each block, 10/26 trails were matches, 4/26 trials matched with the previous but not the symbol two trials ago, and the rest did not match with location of the previous one or two trials. Outcome measures were accuracy and reaction time (RT). In the 0-back condition, participants had to decide whether the presenting symbol matched the position of the first symbol presented in each block. There were two experimental blocks, and the frequency of matches was equivalent to the 2-back conditions.

### Statistical methods

The sleep diary and actigraphy data on the five days before the experimental session were averaged and compared between the Nap- and Wake-group, in addition to the measures on demographic factors (e.g. age, sex, body-mass-index), mood (DASS) and sleep behaviors (PSQI and ESS) by either independent *t*-tests or chi-square tests. The potential group differences on pre-test state-measures (PANAS and SSS) and neurocognitive measures were also analyzed by independent *t*-test.

A 2x2 mixed factorial model was performed on neurocognitive and state-measures with a within-subject factor (time, as the pre- and post-condition performance of the neurocognitive/state-measures) and a between-subject factor (group, Nap-/Wake-group) with any significantly different demographic, mood, sleep as covariates in the analyses. Significantly different working memory measures were further analyzed as follows, A) poor sleep quality (PSQI>5) or excessive daytime sleepiness (ESS>10) were input as an additional between-subject factor into the mentioned 2x2 mixed factorial model to examine whether changes following the daytime sleep opportunity were different among individuals with/without poor sleep quality/daytime sleepiness; B) their correlational relationship with the polysomnographic measures were explored to shed light on potential mechanism of change.

The total sleep time and SOL measured by sleep diary and actigraphy on the night following the experiment were compared by independent *t*-test to explore whether the daytime sleep opportunity would alter subsequent nighttime sleep. Non-parametric analyses were also performed (refer to S1–S4 Tables). Statistical significance was determined by an alpha value of .05.

## Results

While 85 participants satisfied the inclusion (and exclusion criteria), four participants were not included in the final analyses since a) in the nap-group, one participant reported use of caffeinated beverage within 24 hours before the experimental session, and another one failed to fall asleep during the daytime sleep opportunity, and b) in the wake-group, one participant reported use of alcoholic beverage within 24 hours before the experimental session, and another one lost the acti-watch and sleep diary. The final sample then consisted of 81 participants (17–23 years; mean age, 19.91 years; 55.6% females, nap group, n = 40) (S1 and S2 Checklists). Descriptive information of the participants was reported in Table 1. The two groups did not significantly differ on age, sex, body mass index, *p*s>.05. There were also no significant differences on the negative mood as measured by DASS, *p*s>.05.

**Table 1:** Demographics of the sample.

	All (n = 81)	Nap-group (n = 41)	Wake-group (n = 40)	*t* / *x* ^2^	*p*
Age (years)	19.91 (1.4)	19.79 (1.5)	2.03 (1.4)	-.770	.443
Sex (number of males)	36	20	16	.632	.427
Body-mass-index	2.12 (2.7)	2.46 (3.0)	19.78 (2.4)	1.153	.253
Education (years)	14.54 (1.3)	14.45 (1.3)	14.63 (1.4)	-.577	.565
Family income (10K)	4.08 (3.0)	4.06 (2.3)	4.10 (3.7)	-.057	.955
DASS—Depression	4.14 (3.5)	3.76 (2.7)	4.53 (4.2)	-.979	.331
DASS—Anxiety	4.04 (3.3)	3.80 (3.2)	4.28 (3.5)	-.639	.525
DASS—Stress	6.00 (3.8)	5.56 (3.6)	6.45 (4.0)	-1.046	.299

Chi-square test was run only on between-group comparison on sex. Besides sex, *t*, *x* ^2^ and *p*-value, all the figures are mean and standard deviation of each variable. For family income, 10K referred to 10,000 Hong Kong dollars.

The sleep measures derived from sleep diary, actigraphy, PSQI and ESS revealed no significant differences between the groups, *p*s>.05 (Table 2). For total sleep time, both groups reported to have about 7 hours of sleep on average for the five days before the experimental session assessed by both sleep diary and actigraphy. About 43.2% of participants were classified as poor sleepers by PSQI, and 62.0% as having excessive daytime sleepiness by ESS in both groups. In addition, the total sleep time, SOL and WASO were not significantly different between the two groups on the 6^th^ night, after the experimental session, *p*s>.05 (Table 2).

**Table 2:** Sleep-wake patterns of the sample.

	All (n = 81)	Nap-group (n = 41)	Wake-group (n = 40)	*t* / *x* ^2^	*p*
**Sleep diary**					
5-day TST (hour)	7.15 (1.4)	7.01 (1.5)	7.29 (1.3)	-.879	.382
5-day SOL (min)	12.35 (14.3)	14.20 (18.6)	1.50 (7.8)	1.142	.257
5-day WASO (min)	5.76 (11.8)	6.88 (14.1)	4.68 (9.1)	.797	.428
6th day TST (hour)	7.63 (1.5)	7.42 (1.5)	7.86 (1.5)	-1.321	.191
6th day SOL (min)	13.75 (15.7)	11.75 (15.7)	16.03 (18.8)	-1.073	.287
6th day WASO (min)	6.73 (12.1)	5.20 (9.2)	8.49 (14.7)	-1.175	.244
**Actigraphy**					
5-day TST (hour)	7.24(.92)	7.11 (.72)	7.38 (1.1)	-1.018	.314
5-day SOL (min)	5.89 (15.8)	3.03 (7.9)	9.02 (21.1)	-1.297	.201
5-day WASO (min)	15.98 (23.4)	13.20 (8.2)	19.02 (32.8)	-.841	.405
6th day TST (hour)	7.17 (1.7)	6.80 (1.8)	7.51 (1.5)	-1.673	.100
6th day SOL (min)	11.53 (13.6)	2.31 (5.9)	4.19 (14.6)	1.730	.089
6th day WASO (min)	3.30 (11.3)	14.66 (17.0)	8.71 (9.0)	-.646	.521
**PSQI**	6.30 (2.9)	6.32 (3.0)	6.28 (2.7)	.054	.957
PSQI—poor sleepers(n)	35	16	19	.593	.441
**ESS**	11.25 (4.3)	1.58 (4.0)	11.90 (4.6)	-1.372	.174
ESS—excessive daytime sleepiness (n)	49	24	25	.008	.930

TST = total sleep time; SOL = sleep onset latency; WASO = duration of wake after sleep onset; PSQI = Pittsburgh Sleep Quality Index; ESS = Epworth Sleepiness Scale. Excessive daytime sleepiness referred to an ESS>1. Chi-square test was run only on between-group comparison on PSQI-poor sleepers and ESS-excessive daytime sleepiness. Besides PSQI-poor sleepers and ESS-excessive daytime sleepiness, *t*, *x* ^2^ and *p*-value, all the figures are mean and standard deviation of each variable.

### Impact of a daytime sleep opportunity on working memory ability

During the pre-condition session, participants in both group did not differ on the SSS, the PANAS, as well as the PVT, *p*s>.05. The groups also did not significantly differ on the RT and accuracy measures of the 0-back and 2-back conditions of the n-back task (*p*s>.05). Given that there were no significant differences on the groups’ baseline performance on all measures assessed, no covariates were entered in the mixed factorial analyses.

On the state-measures (Table 3), results from the factorial model showed a significant interaction effect of time and group on state-sleepiness (SSS score), *F*_2,79_ = 9.590, *p* = .003, η^2^ = .109. Post-hoc analyses (paired-sample *t*-test) showed that while the Nap-group had decreased SSS across time, *t*(39) = 2.639, *p* = .012, the changes in the Wake-group were not significant, *t*(39) = -1.657, *p* = .105. For positive affect, there was a significant interaction effect of time and group, *F*_2,79_ = 6.103, *p* = .016, η^2^ = .073. Post-hoc analyses (paired-sample *t*-test) showed that significant decrease in positive affect on PANAS post-condition was only shown in the Wake-group, *t*(39) = 2.739, *p* = .009 and not the Nap-group, *t*(39) = -.704, *p* = .486. On negative affect, there was a significant main effect of time on PANAS-negative affect score, *F*_1,80_ = 26.393, *p*<.001, η^2^ = .253, in which both Nap- and Wake-group had significantly lower PANAS-negative affect score in the post-condition assessment. On psychomotor vigilance, there was a significant interaction effect between time and group on the average response time, *F*_2,65_ = 1.799, *p* = .002, η^2^ = .142 and 1/response time, *F*_2,65_ = 18.569, *p*<.001, η^2^ = .222. Post-hoc analyses (paired-sample *t*-test) showed that while the Nap-group had faster PVT average response time, *t*(35) = 3.004, *p* = .005 and 1/response time, *t*(35) = -3.471, *p* = .001, in the post-condition assessment, the Wake-group’s PVT average response time did not significantly change, *t*(30) = -1.654, *p* = .109, and were noted with slower 1/response time, *t*(30) = 2.722, *p* = .011. There was no significant main or interaction effect on the PVT lapses, *p*s>.05.

**Table 3:** Between-group comparisons on state-measures.

	Nap-group (n = 41)		Wake-group (n = 40)		*F*_*time*_	*F*_*group*_	*F*_*time* x group_
	Pre	Post	Pre	Post			
SSS	2.88 (.94)	2.33 (.72)	2.43 (.71)	2.68 (.92)	1.066	<.001	9.590**
PANAS—Positive	26.55(5.5)	27.08 (6.1)	26.85 (5.8)	24.70 (6.9)	2.252	.686	6.103*
PANAS—Negative	16.78(5.0)	13.83 (4.6)	16.55 (5.6)	14.73 (5.8)	26.393***	.096	1.465
PVT—RT	.29 (.04)	.28 (.03)	.28 (.03)	.29 (.04)	1.066	.027	1.799**
PVT—1/RT	3.70 (.33)	3.82 (.35)	3.82 (.34)	3.69 (.38)	.060	.001	18.569***
PVT—lapses	2.81 (3.0)	2.83 (2.6)	3.00 (2.4)	3.58 (4.1)	.544	.551	.449

SSS = Stanford Sleepiness Scale; PANAS-Positive/Negative = Positive and Negative Affect Schedule positive/negative score; PVT = Psychomotor Vigilance Task; RT = reaction time. Apart from F-values, all other figures are mean and standard deviation of each variable.

**p<.01,

***p<.001.

**Fig 1:**
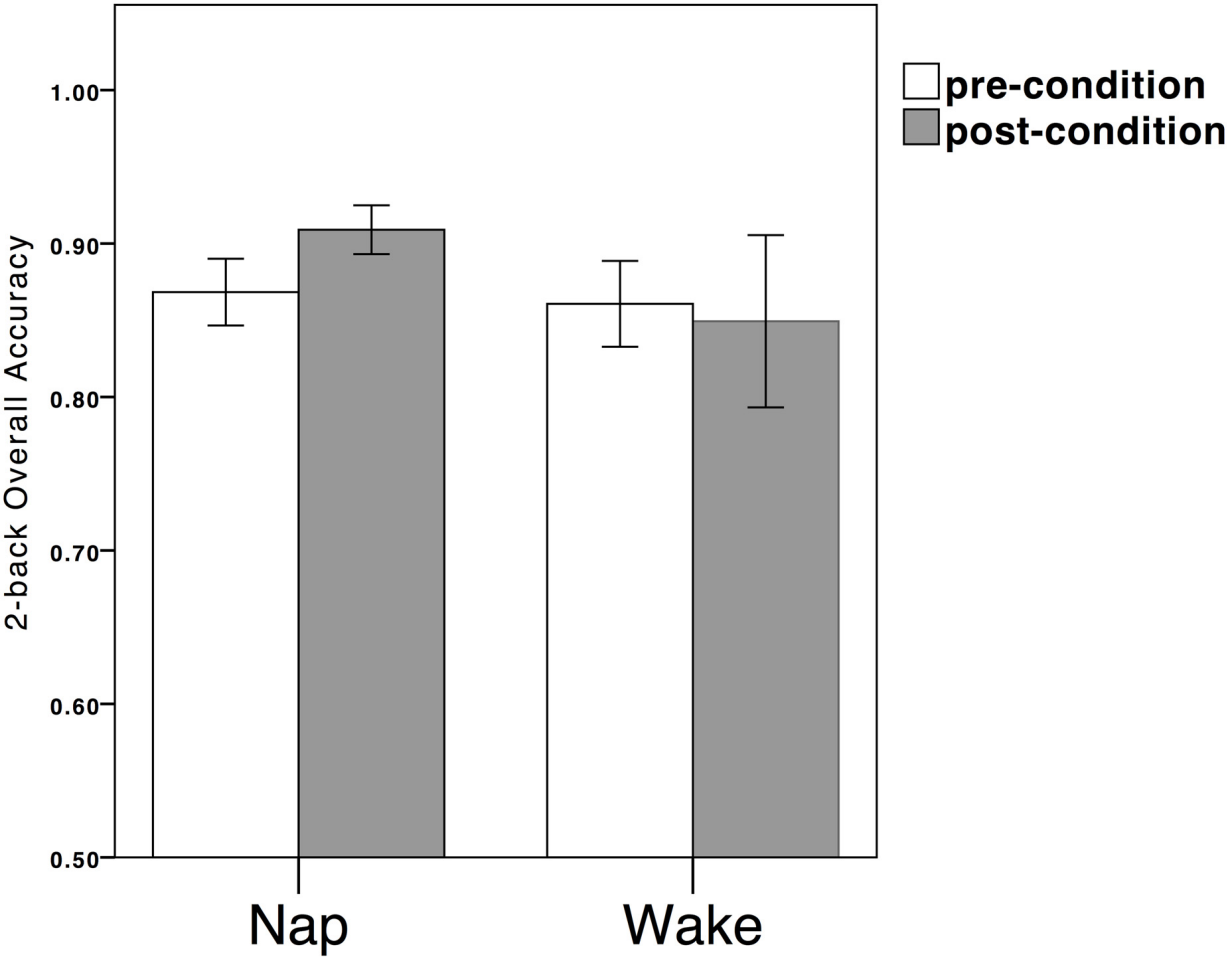
Significant improvement on the 2-back overall accuracy at posttest for the Nap-group.

On the N-back task (Table 4), 0-back condition, which measured basic attention, there were no statistically significant results observed. On the 2-back task, which measured working memory ability, there was a significant interaction effect of condition and time on the overall accuracy measure, *F*_2,73_ = 4.289, *p* = .042, η^2^ = .055. Post-hoc analysis (paired-sample *t*-test) showed that while the Nap-group had improved overall accuracy on the 2-back task post-condition, *t*(39) = -4.021, *p*<.001, cohen’s *d* = -1.272, there was no significant difference in the 2-back accuracy across time among the Wake-group, *t*(33) = .664, *p* = .511 (Fig 1). To be specific, there was a significant interaction effect between time and condition in the second block, *F*_2,73_ = 5.905, *p* = .017, η^2^ = .073, and third block, *F*_2,73_ = 4.476, *p* = .038, η^2^ = .058, of the 2-back task. Post-hoc analyses (paired-sample *t*-tests) indicated that while the Nap-group had significantly improved accuracy in block 2, *t*(39) = -2.939, *p* = .005, and block 3, *t*(39) = -2.092, *p* = .041, in the post-condition, there were no significant changes of accuracy in block 2, *t*(35) = .982, *p* = .333, or block 3, *t*(33) = 1.495, *p* = .144, among the Wake-group. On the response time measure, there was a significant main effect of time on the overall RT, *F*_1,74_ = 43.669, *p*<.001, block 1, *F*_1,77_ = 38.567, *p*<.001, block 2, *F*_1,76_ = 33.139, *p*<.001, and block 3, *F*_1,74_ = 14.930, *p*<.001, in which both groups had faster response time in all the blocks in post-condition assessment.

**Table 4:** Between-group comparisons on basic attention and working memory ability.

	Nap-group (n = 40)		Wake-group (n = 41)		*F*_*time*_	*F*_*group*_	*F*_*time* x *group*_
	Pre	Post	Pre	Post			
**Basic Attention (0-back)**							
Acc	.93 (.05)	.93 (.06)	.93 (.07)	.90(.14)	.784	1.689	.784
RT	.49 (.08)	.53 (.10)	.51 (.11)	.51(.11)	2.600	<.001	2.342
**Working Memory (2-back)**							
Overall Acc	.87 (.07)	.91 (.05)	.86 (.08)	.84(.17)	.519	3.716	4.289*
Block 1 Acc	.87 (.10)	.92 (.06)	.85 (.13)	.87(.17)	3.326	2.784	.375
Block 2 Acc	.85 (.10)	.91 (.09)	.87 (.09)	.84(.18)	.498	1.237	5.905*
Block 3 Acc	.89 (.09)	.91 (.07)	.88 (.11)	.84(.18)	.527	3.303	4.476*
Overall RT	.95 (.27)	.83 (.25)	.97 (.29)	.85(.27)	43.669***	.210	.156
Block 1 RT	.96 (.33)	.85 (.29)	1.05(.33)	.85(.26)	38.567***	1.071	1.189
Block 2 RT	.96 (.30)	.81 (.27)	.97(.29)	.86(.31)	33.139***	.225	1.084
Block 3 RT	.94 (.27)	.83 (.25)	.92(.28)	.85(.28)	14.930***	.001	1.171

0-back = N-back task, 0-back condition; 2-back = N-back task, 2-back condition; Acc = Accuracy; RT = reaction time (seconds). Apart from F-values, all other figures are mean and standard deviation of each variable.

*p<.05,

***p<.001.

### The role of poor sleep in the relationship between daytime sleep opportunity and working memory ability

Results from the 2(condition)x2(time)x2(poor sleep, either poor sleep quality or excessive daytime sleepiness) factorial model showed no significant interaction effect among poor sleep quality with condition and time on the 2-back overall accuracy, *F*_3,72_ = .058, *p* = .810, second block accuracy, *F*_3,74_ = 1.095, *p* = .299, or third block accuracy, *F*_3,72_ = .588, *p* = .446. Also, excessive daytime sleepiness did not interact with condition and time on the 2-back overall accuracy, *F*_3,70_ = 2.284, *p* = .135, second block accuracy, *F*_3,71_ = .530, *p* = .469, or third block accuracy, *F*_3,70_ = 2.042, *p* = .158.

### Relationship between working memory and polysomnographic measures

**Fig 2:**
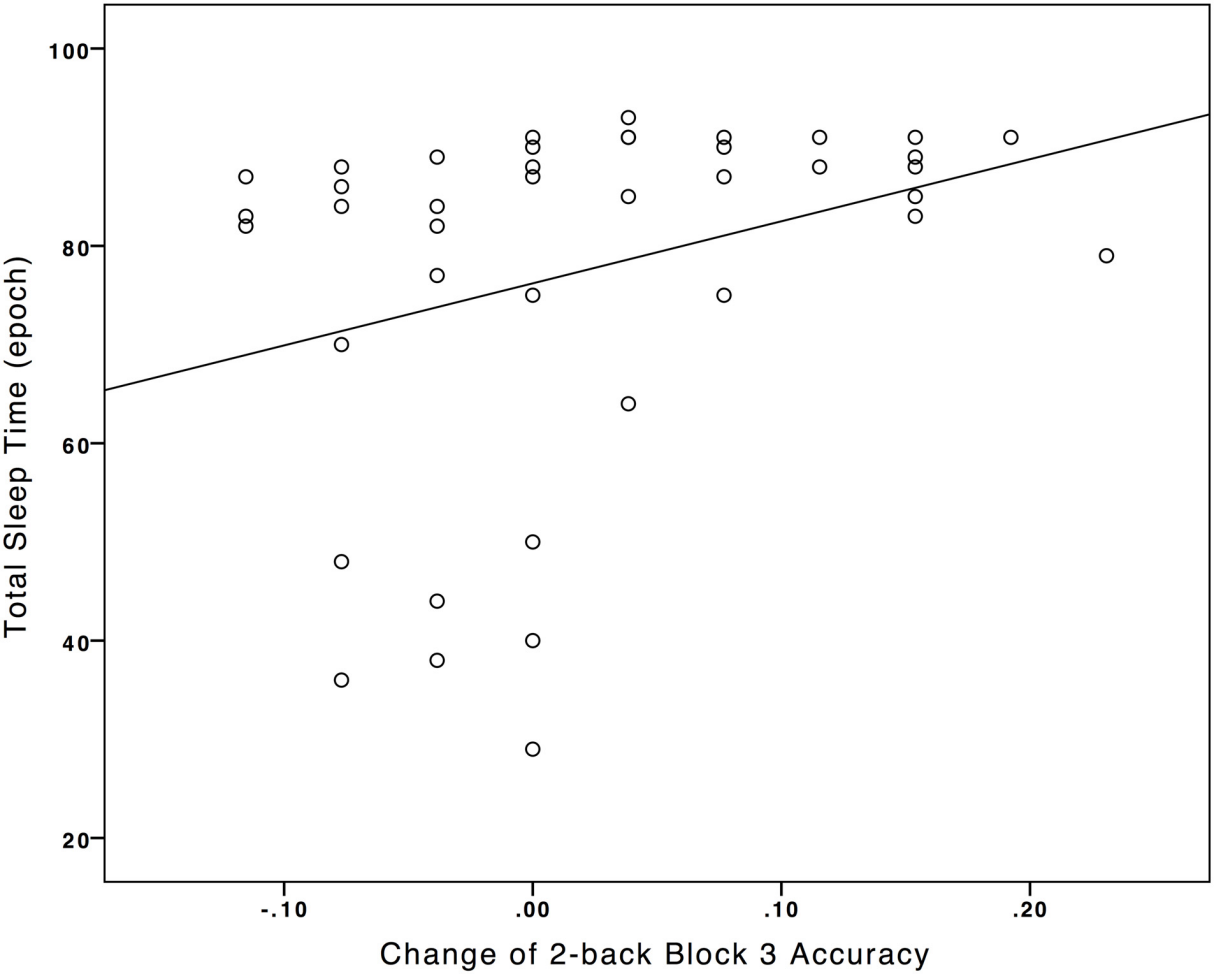
Association between total-sleep-time during napping with the pre/post-condition difference of 2-back Block 3 Accuracy.

**Fig 3:**
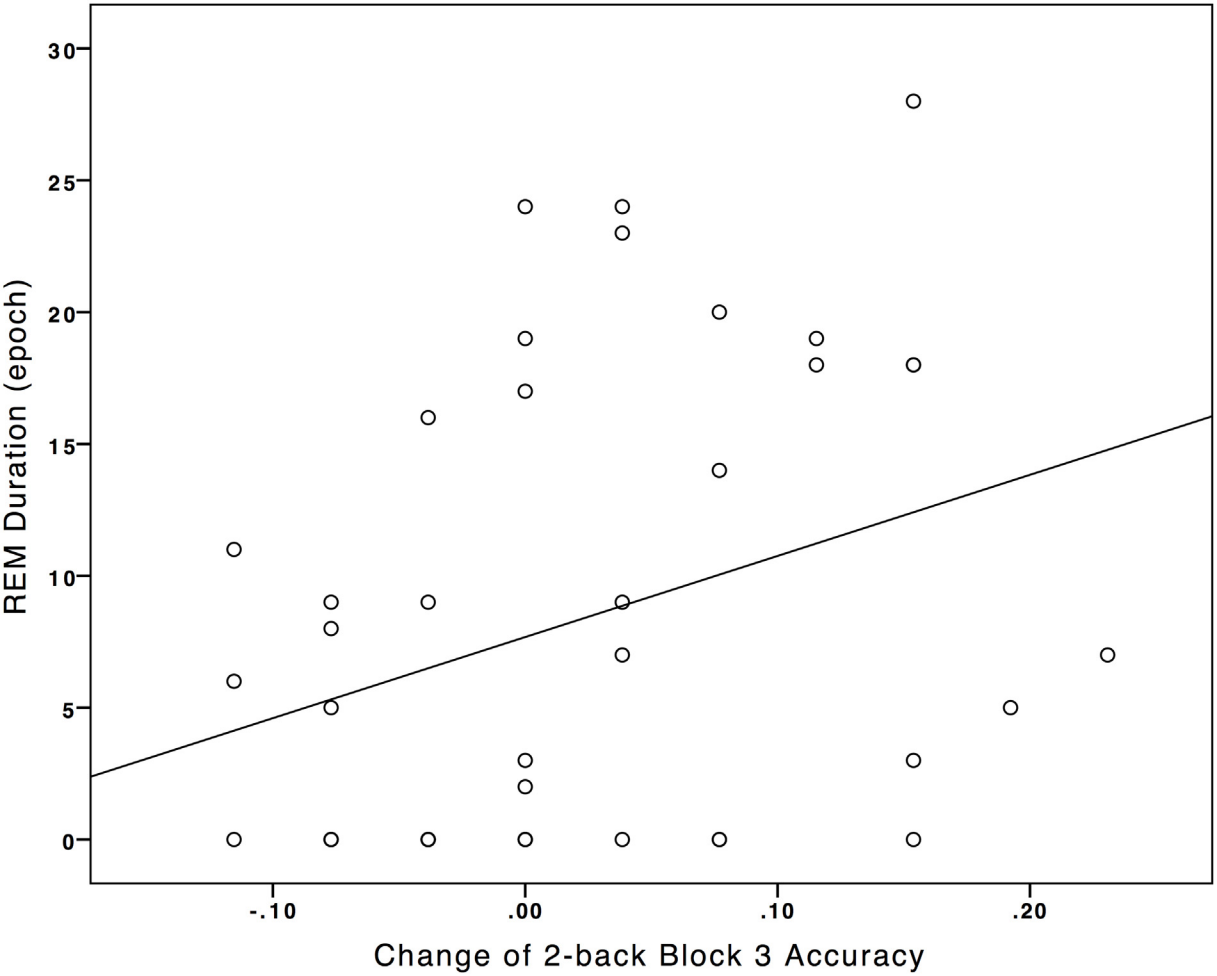
Association between Rapid-eye-movement-sleep duration with the pre/post-condition difference of 2-back Block 3 Accuracy.

As the Nap-group was found to have significant improvement in the accuracy on the 2-back task across time, the association between the change in accuracy on the 2-back (from pre- to post-condition) and the polysomnographic measures were studied by correlational analyses. Details of the polysomnographic data were reported in Table 5. There were significant positive correlations between the change of 2-back block 3 accuracy with the total sleep time during the nap, *r*(40) = .315, *p* = .045 (Fig 2) and duration of REM, *r*(32) = .319, *p* = .042 (Fig 3). No other significant relationship was noted (Table 6).

**Table 5:** Polysomnographic data of the Nap-group.

	Mean (standard deviation)
Total Sleep Time	77.56 min (18.18)
Sleep efficiency	78.03% (19.29)
Wake after Sleep Onset	9.80 min (12.58)
Sleep Onset Latency	8.83 min (6.56)
REM latency	64.31 min (9.77)
REM	8.34 min (8.78)
REM %	9.34% (9.62)
N1	12.54 min (8.17)
N1%	16.71% (1.64)
N2	4.10 min (13.06)
N2%	53.57% (15.08)
SWS	16.50 min (13.43)
SWS %	19.63% (15.79)

REM = Rapid eye movement sleep.

N1 = Non-rapid eye movement stage 1 sleep.

N2 = Non-rapid eye movement stage 2 sleep.

SWS = slow wave sleep.

**Table 6:** Correlational relationships between polysomnographic data and pre-post condition changes on working memory measures among the Nap-group.

	Overall Acc	B1 Acc	B2 Acc	B3 Acc	Overall RT	B1 RT	B2 RT	B3 RT
TST	.164	-.013	.039	.315*	-.186	.014	-.173	-.029
SE	.138	-.002	.009	.284	-.129	.035	-.151	.028
WASO	-.105	.056	-.026	-.258	.235	-.006	.209	-.019
SOL	.047	-.059	.082	.066	-.248	-.234	-.037	-.272
REM latency	-.038	.276	-.134	-.268	.002	-.112	.186	-.008
REM	.106	-.216	.132	.319*	.001	-.001	-.047	.12
REM %	.112	-.224	.153	.314*	.011	.006	-.033	.121
N1	.141	-.062	.192	.125	.088	.181	-.043	-.076
N1%	.05	-.065	.163	-.027	.121	.113	-.024	-.101
N2	.14	.133	.145	-.052	-.11	.021	-.137	-.007
N2%	.015	.119	.135	-.298	-.001	-.055	.004	.011
SWS	-.025	.054	-.245	.182	-.211	-.159	-.06	-.023
SWS %	-.048	.069	-.269	.145	-.161	-.133	-.006	.003

REM = Rapid eye movement sleep; N1 = Non-rapid eye movement stage 1 sleep.

N2 = Non-rapid eye movement stage 2 sleep; SWS = slow wave sleep.

TST = total sleep time; SE = sleep efficiency; WASO = wake duration after sleep onset.

SOL = sleep onset latency; Acc = accuracy; B1 = block 1; B2 = block 2; B3 = block 3.

RT = reaction time.

*p<.05.

## Discussion

The primary aim of the study was to investigate the relationship between sleep and working memory ability, as well as the mechanism behind such relationship, with the use of a daytime sleep opportunity. To the best knowledge of the authors, this was the first study reporting that without manipulation of sleep-wake patterns prior to the experimental protocol, healthy adults with poor sleep characteristics (43.2% poor sleeper by PSQI, 62.0% excessive daytime sleepiness by ESS) had improved working memory ability following a daytime sleep opportunity, relative to wakefulness. The findings were also first to document an association between working memory ability and REM duration and total sleep time in the nap among healthy adults.

### Working memory enhancement following a daytime sleep opportunity

Consistent with our first hypothesis, participants provided with a daytime sleep opportunity showed improved accuracy in the working memory task and such improvement was not observed in the participants who did not have the sleep opportunity and was also not dependent on individuals’ sleep quality or daytime sleepiness at baseline. Our results were consistent with existing literatures using sleep deprivation or chronic sleep restriction paradigms. For instance, with a similar working memory task, Smith and coworkers [5] found that sleep-deprived participants had worse working memory ability than well-rested healthy adults. Lo and coworkers [4] also found chronically sleep-restricted adults had worse working memory ability than well-rested adults. Collectively, these data supported the essential role of sleep in working memory ability. Indeed, the medium to large effect sizes (η^2^ = .055, *d* = -1.272) observed in the current study suggest that working memory ability may be sensitive to sleep gain during the day. The current study also distinguished itself from previous studies by the generalizability of its findings. Firstly, since we did not manipulate participants’ sleep-wake patterns before testing, and participants were instead asked to follow their habitual sleep-wake schedules throughout the experimental protocols, it was expected the findings from the current study had higher ecological validity. Indeed, the habitual sleep-wake patterns of our sample also appeared to be similar to other studies. In our previous longitudinal study, the young adult sample (n = 930) reported to have about 7 hours of nighttime sleep, similar to our current sample [38]. Secondly, while existing napping studies mostly included either habitual nappers [39] or non-habitual nappers [40], both types of participants were included in the Nap/Wake-group so that the findings would not be solely applicable to individuals with/without habitual napping behaviors. Thirdly, it should be noted that participants in the Nap-/Wake-group reported to have similar total sleep time, SOL and WASO from sleep diary and actigraphy on the night following the experimental session, which suggested that a daytime sleep opportunity within 90 minutes did not significantly lead to worse nighttime sleep behaviors when compared to those in the Wake-group. Yet, it should be noted that the mean total sleep time of our sample was about 7 hours (range from 4.21 to 9.27 hours), and the current findings could not be directly applied to individuals with different habitual total sleep time. Fourthly, the use of both self-report (sleep diary) and objective measures (actigraph-watch) of habitual sleep wake behaviors enhanced the reliability and validity of our findings.

### Sleep physiology and working memory

Total sleep time during a daytime nap and REM duration were found to positively correlate with performance in the third block of the working memory task among the Nap-group. From the first to the third block, the load on working memory conceivably increased as participants needed to sustain their attention to process, store and manipulate the learnt information (spatial location of the stimuli). The results might therefore indicate that with increasing load on working memory, the correlation between working memory function with REM and total sleep time might become apparent.

The association between total sleep time during the nap with working memory ability was indeed consistent with existing literature studying the effect of sleep loss on working memory performance in which sufficient sleep was vital to working memory performance [4, 15, 18]. The current study added to existing knowledge that not only sleep loss, but “sleep gain” from a daytime sleep opportunity of 90 minutes was observed to have significant correlation with working memory, among the study sample, who had habitual short sleep duration (7 hours) and poor sleep quality, which were common among young adults nowadays [38].

The relationship between duration of REM with working memory could be interpreted with reference to the changes of neural activities of the PFC during REM. Existing literatures showed that the PFC activation and functional connectivity with other brain regions decreased from wake to NREM, but increased again during REM [20–24]. For example, Kubota and coworkers [25] recent neuroimaging data showed that the onset of REM (REM latency) co-occurred with the re-activation of dorsolateral PFC during sleep. While the exact reason behind the dynamic changes of PFC neural activities from NREM to REM remained unknown, REM appeared to have an important role regarding the selective activation and deactivation of PFC during sleep. We therefore speculated that enhanced working memory could be a behavioral manifestation of optimal PFC neural activities, which could be preserved by sufficient REM. In addition, abnormality in REM was indeed consistently observed in patients with psychiatric and medical conditions, e.g. major depressive disorder [41], narcolepsy [42, 43], which mostly presented with both sleep complaints and executive functions deficits. It then became interesting and worthwhile to study whether executive dysfunctions among healthy and/or patient populations were related to disrupted sleep architecture, or even specifically related to disrupted REM physiology during sleep. In a recent study conducted among healthy adults being exposed to high altitude location, shorter REM latency was also found to correlate with worse working memory among those with hypoxic condition (but not in normoxic condition) [44]. While our study was one of the first studies observing a positive correlation between REM and working memory performance after a daytime sleep opportunity, we call for future studies to assess the relationship between other higher-ordered cognitive functions subserved by the PFC with REM and other sleep physiological measures among healthy and patient populations to help elucidate the relation between sleep and higher ordered cognitive functions as well as the mechanisms behind.

There were several shortcomings of the current study which needed to be considered. First, while the mean total sleep time of our sample was about 7 hours, nearly half of them had poor sleep quality (43.2% with PSQI global score>5) and nearly two-third reporting excessive daytime sleepiness (62.0% with ESS>10), we could not ascertain whether the benefit of daytime sleep opportunity would apply to individuals with longer/shorter habitual total sleep time or better/worse sleep quality. Yet, such total sleep time was similar to what was reported among this age group in previous studies [38]. In addition, our results also showed that poor sleep (e.g. poor sleep quality and excessive daytime sleepiness) did not significantly moderate the effect of daytime sleep opportunity on the working memory measures, which potentially indicate that “sleep gain” during the daytime could be benefiting working memory performance among individuals with/without poor sleep quality/excessive daytime sleepiness. We called forfuture studies investigating the role of daytime sleep opportunity on individuals with different sleep-wake patterns to further verify the generalizability of our findings. Secondly, we only made use of one working memory measure so the effects of napping on working memory measured in other ways are yet to be elucidated. Nevertheless, the n-back task was widely used in the sleep literature, including sleep deprivation [4], chronic sleep restriction [18] or sleep-disordered patient [11] studies and was consistently found to be a valid measure of working memory and a sensitive measure of sleep loss. Thirdly, with the current research design, only correlational but not causal relationship could be inferred between REM and changes in working memory performance after a daytime sleep opportunity.

Taken together, the current results of the benefits of a daytime nap on working memory were in line with the literature on sleep loss and related impact on PFC functioning and working memory deficits. We also extended existing understanding by observing a correlation between REM duration and total sleep time and enhanced working memory ability following a daytime nap. Such findings were complementary to studies on executive function deficits following sleep loss or among patients with psychiatric or medical conditions associated with abnormal REM physiology. The observation that the napping manipulation did not disrupt subsequent night time sleep further enhanced the applicability of napping in future experiments, daily life and potential clinical use.

## Supporting Information

S1 Checklist CONSORT 2010 Flow Diagram checklist. (DOC)

S2 Checklist CONSORT 2010 Checklist. (DOC)

S1 Table Group difference on demographics (non-parametric analyses). (DOCX)

S2 Table Sleep-wake patterns of the sample (non-parametric analyses). (DOCX)

S3 Table Between-group comparisons on state-measures (non-parametric analyses). (DOCX)

S4 Table Between-group comparisons on basic attention and working memory ability (non-parametric analyses). (DOCX)
