# Supplementary material for: Rapid-Eye-Movement-Sleep (REM) Associated Enhancement of Working Memory Performance after a Daytime Nap
Source: PLoS One. 2015 May 13;10(5):e0125752. doi: 10.1371/journal.pone.0125752 (PMC4430242; doi:10.1371/journal.pone.0125752)
Supplement: S1 Checklist — (DOC) [file pone.0125752.s001.doc]

**
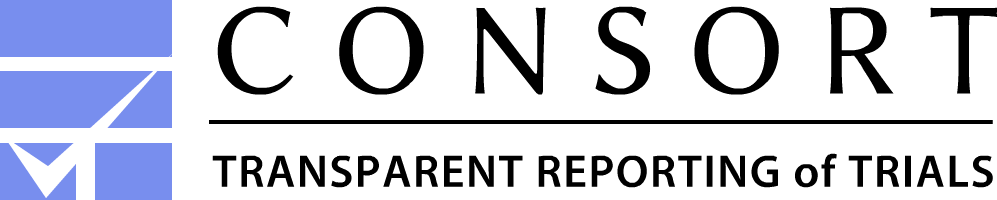
**

**S1 Checklist – CONSORT 2010 Flow Diagram**

**Allocation**

**Analysis**

**Follow-Up**

**Enrollment**

Assessed for eligibility (n=90)

Excluded (n=5)

♦  Not meeting inclusion criteria (n=5)

♦  Declined to participate (n=0)

♦  Other reasons (n=0)

Analysed (n=40)
♦ Excluded from analysis (give reasons) (n=2) (1 due to use of caffeinated beverage within 24 hours before the experimental session; 1 due to failure to fall asleep during the daytime sleep opportunity)

Lost to follow-up (give reasons) (n=0)

Discontinued intervention (give reasons) (n=0)

Allocated to intervention (n=43)

♦ Received allocated intervention (n=43)

♦ Did not receive allocated intervention (n=0)

Lost to follow-up (give reasons) (n=0)

Discontinued intervention (give reasons) (n=0)

Allocated to intervention (n=42)

♦ Received allocated intervention (n=42)

♦ Did not receive allocated intervention (n=0)

Analysed (n=40)
♦ Excluded from analysis (n=2) (1 due to loss of acti-watch,, another due to use of alcoholic beverage within 24 hours before the experimental session

Randomized (n=85)
