# Supplementary material for: Rapid-Eye-Movement-Sleep (REM) Associated Enhancement of Working Memory Performance after a Daytime Nap
Source: PLoS One. 2015 May 13;10(5):e0125752. doi: 10.1371/journal.pone.0125752 (PMC4430242; doi:10.1371/journal.pone.0125752)
Supplement: S1 Table — (DOCX) [file pone.0125752.s003.docx]

**S1 Table** – Group difference on demographics (non-parametric analyses)

|  | All (n=81) | Nap-group (n=41) | Wake-group (n=40) | *U* / *x*^2^ | *p* |
| --- | --- | --- | --- | --- | --- |
| Age (years) | 19.91 (1.4) | 19.79 (1.5) | 2.03 (1.4) | 710 | .297 |
| Sex (number of males) | 36 | 20 | 16 | .632 | .427 |
| Body-mass-index | 2.12 (2.7) | 2.46 (3.0) | 19.78 (2.4) | 740 | .450 |
| Education (years) | 14.54 (1.3) | 14.45 (1.3) | 14.63 (1.4) | 751 | .498 |
| Family income (10K) | 4.08 (3.0) | 4.06 (2.3) | 4.10 (3.7) | 723 | .356 |
| DASS – Depression | 4.14 (3.5) | 3.76 (2.7) | 4.53 (4.2) | 803 | .868 |
| DASS – Anxiety | 4.04 (3.3) | 3.80 (3.2) | 4.28 (3.5) | 763 | .588 |
| DASS – Stress | 6.00 (3.8) | 5.56 (3.6) | 6.45 (4.0) | 722 | .350 |

Chi-square test was run only on between-group comparison on sex. Besides sex, *U*-, *x*^2^ and *p*-value, all the figures are mean and standard deviation of each variable. For family income, 10K referred to 10,000 Hong Kong dollars.
