# Supplementary material for: Rapid-Eye-Movement-Sleep (REM) Associated Enhancement of Working Memory Performance after a Daytime Nap
Source: PLoS One. 2015 May 13;10(5):e0125752. doi: 10.1371/journal.pone.0125752 (PMC4430242; doi:10.1371/journal.pone.0125752)
Supplement: S2 Table — (DOCX) [file pone.0125752.s004.docx]

**S2 Table** – Sleep-wake patterns of the sample (non-parametric analyses)

|  | All (n=81) | Nap-group (n=41) | Wake-group  (n=40) | *U* / *x*^2^ | *p* |
| --- | --- | --- | --- | --- | --- |
| **Sleep diary** |  |  |  |  |  |
| 5-day TST (hour) | 7.15 (1.4) | 7.01 (1.5) | 7.29 (1.3) | 581 | .103 |
| 5-day SOL (min) | 12.35 (14.3) | 14.20 (18.6) | 1.50 (7.8) | 690 | .478 |
| 5-day WASO (min) | 5.76 (11.8) | 6.88 (14.1) | 4.68 (9.1) | 649 | .824 |
| 6th day TST (hour) | 7.63 (1.5) | 7.42 (1.5) | 7.86 (1.5) | 505 | .077 |
| 6th day SOL (min) | 13.75 (15.7) | 11.75 (15.7) | 16.03 (18.8) | 640 | .517 |
| 6th day WASO (min) | 6.73 (12.1) | 5.20 (9.2) | 8.49 (14.7) | 615 | .307 |
| **Actigraphy** |  |  |  |  |  |
| 5-day TST (hour) | 7.24(.92) | 7.11 (.72) | 7.38 (1.1) | 184 | .079 |
| 5-day SOL (min) | 5.89 (15.8) | 3.03 (7.9) | 9.02 (21.1) | 230 | .379 |
| 5-day WASO (min) | 15.98 (23.4) | 13.20 (8.2) | 19.02 (32.8) | 235 | .524 |
| 6th day TST (hour) | 7.17 (1.7) | 6.80 (1.8) | 7.51 (1.5) | 351 | .101 |
| 6th day SOL (min) | 11.53 (13.6) | 2.31 (5.9) | 4.19 (14.6) | 365 | .150 |
| 6th day WASO (min) | 3.30 (11.3) | 14.66 (17.0) | 8.71 (9.0) | 446 | .698 |
| **PSQI** | 6.30 (2.9) | 6.32 (3.0) | 6.28 (2.7) | 784 | .881 |
| PSQI –poor sleepers(n) | 35 | 16 | 19 | .593 | .441 |
| **ESS** | 11.25 (4.3) | 1.58 (4.0) | 11.90 (4.6) | 631 | .141 |
| ESS – excessive daytime sleepiness (n) | 49 | 24 | 25 | .008 | .930 |

TST=total sleep time; SOL=sleep onset latency; WASO=duration of wake after sleep onset; PSQI=Pittsburgh Sleep Quality Index; ESS=Epworth Sleepiness Scale. Excessive daytime sleepiness referred to an ESS>1. Chi-square test was run only on between-group comparison on PSQI-poor sleepers and ESS-excessive daytime sleepiness. Besides PSQI-poor sleepers, ESS-excessive daytime sleepiness, *U*-, *x*^2^ and *p*-value, all the figures are mean and standard deviation of each variable.
