# Supplementary material for: Rapid-Eye-Movement-Sleep (REM) Associated Enhancement of Working Memory Performance after a Daytime Nap
Source: PLoS One. 2015 May 13;10(5):e0125752. doi: 10.1371/journal.pone.0125752 (PMC4430242; doi:10.1371/journal.pone.0125752)
Supplement: S3 Table — (DOCX) [file pone.0125752.s005.docx]

**S3 Table** – Between-group comparisons on state-measures (non-parametric analyses)

|  | Nap-group  (n=40) | | Wake-group  (n=41) | | *Z_time_* | *U_pre_* | *U_post_* |
| --- | --- | --- | --- | --- | --- | --- | --- |
|  | Pre | Post | Pre | Post |  |  |  |
| SSS | 2.88  (.94) | 2.33 (.72) | 2.43  (.71) | 2.68 (.92) | -.868 | 620 | 640 |
| PANAS-Positive | 26.55  (5.5) | 27.08 (6.1) | 26.85(5.8) | 24.70 (6.9) | -1.391 | 755 | 634 |
| PANAS-Negative | 16.78(5.0) | 13.83 (4.6) | 16.55 (5.6) | 14.73 (5.8) | -5.148*** | 768 | 719 |
| PVT–RT | .29 (.04) | .28 (.03) | .28 (.03) | .29 (.04) | -1.565 | 476 | 468 |
| PVT–1/RT | 3.70  (.33) | 3.82 (.35) | 3.82  (.34) | 3.69 (.38) | -.493 | 474 | 459 |
| PVT–lapses | 2.81  (3.0) | 2.83 (2.6) | 3.00 (2.4) | 3.58 (4.1) | -.309 | 496 | 552 |

SSS=Stanford Sleepiness Scale; PANAS-Positive/Negative=Positive and Negative Affect Schedule positive/negative score; PVT=Psychomotor Vigilance Task; RT=reaction time. Apart from Z- and U-values, all other figures are mean and standard deviation of each variable; ****p*<.001.
