# Supplementary material for: Rapid-Eye-Movement-Sleep (REM) Associated Enhancement of Working Memory Performance after a Daytime Nap
Source: PLoS One. 2015 May 13;10(5):e0125752. doi: 10.1371/journal.pone.0125752 (PMC4430242; doi:10.1371/journal.pone.0125752)
Supplement: S4 Table — (DOCX) [file pone.0125752.s006.docx]

**S4** **Table** – Between-group comparisons on basic attention and working memory ability (non-parametric analyses)

|  | Nap-group  (n=40) | | Wake-group  (n=41) | | *Z_time_* | *U_pre_* | *U_post_* |
| --- | --- | --- | --- | --- | --- | --- | --- |
|  | Pre | Post | Pre | Post |  |  |  |
| **Basic Attention (0-back)** | | | | |  |  |  |
| Acc | .93 (.05) | .93 (.06) | .93 (.07) | .90 (.14) | -.440 | 673 | 644 |
| RT | .49 (.08) | .53 (.10) | .51 (.11) | .51 (.11) | -1.384 | 669 | 618 |
| **Working Memory (2-back)** | | | |  |  |  |  |
| Overall Acc | .87 (.07) | .91 (.05) | .86 (.08) | .84 (.17) | -3.404** | 745 | 522* |
| Block 1 Acc | .87 (.10) | .92 (.06) | .85 (.13) | .87 (.17) | -3.181** | 761 | 712 |
| Block 2 Acc | .85 (.10) | .91 (.09) | .87 (.09) | .84 (.18) | -2.339* | 732 | 555* |
| Block 3 Acc | .89 (.09) | .91 (.07) | .88 (.11) | .84 (.18) | -.296 | 774 | 530* |
| Overall RT | .95 (.27) | .83 (.25) | .97 (.29) | .85 (.27) | -5.703*** | 727 | 697 |
| Block 1 RT | .96 (.33) | .85 (.29) | 1.05(.33) | .85 (.26) | -5.038*** | 692 | 721 |
| Block 2 RT | .96 (.30) | .81 (.27) | .97(.29) | .86 (.31) | -5.242*** | 776 | 688 |
| Block 3 RT | .94 (.27) | .83 (.25) | .92(.28) | .85 (.28) | -3.723*** | 777 | 680 |

0-back=N-back task, 0-back condition; 2-back=N-back task, 2-back condition; Acc=Accuracy; RT=reaction time (seconds). Apart from Z and U-values, all other figures are mean and standard deviation of each variable; **p*<.05, ***p*<.01,****p*<.001
